# Aqua­bis(benzoato-κ*O*)(5,5′-dimethyl-2,2′-bipyridine-κ^2^ *N*,*N*′)copper(II)

**DOI:** 10.1107/S1600536809039191

**Published:** 2009-10-03

**Authors:** Xi-Yan Dong, Xiaojie Xu, Lei Yang

**Affiliations:** a Department of Physics and Chemistry, Henan Polytechnic University, Jiaozuo 454000, Henan, People’s Republic of China

## Abstract

In the crystal structure of the title compound, [Cu(C_7_H_5_O_2_)_2_(C_12_H_12_N_2_)(H_2_O)], the Cu^II^ ion is penta­coordinated in a distorted square-pyramidal geometry by two O atoms of two benzoate anions and two N atoms of a 5,5′-dimethyl-2,2′-bipyridine ligand occupying the basal plane, and a water O atom located at the apical site. In the crystal structure, O—H⋯O hydrogen bonds link the mol­ecules into a supra­molecular structure. The crystal studied was a racemic twin, as suggested by the Flack parameter of 0.584 (14).

## Related literature

For related structures, see: Zhao & Bai (2009 ▶); Schubert, Eschbaumer *et al.* (1999 ▶); Schubert, Hochwimmer *et al.* (1999 ▶); Shi(2009 ▶); Zhang *et al.* (2009 ▶); Momeni *et al.* (2009 ▶); Kim *et al.* (2009 ▶); Yang *et al.* (2001 ▶).
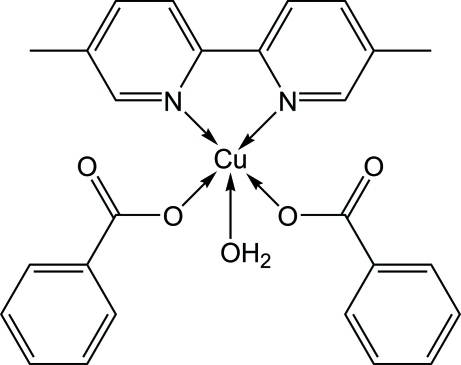


## Experimental

### 

#### Crystal data

[Cu(C_7_H_5_O_2_)_2_(C_12_H_12_N_2_)(H_2_O)]
*M* *_r_* = 508.01
Orthorhombic, 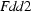

*a* = 36.033 (6) Å
*b* = 37.681 (6) Å
*c* = 7.0402 (12) Å
*V* = 9559 (3) Å^3^
*Z* = 16
Mo *K*α radiation
μ = 0.95 mm^−1^
*T* = 296 K
0.20 × 0.18 × 0.16 mm

#### Data collection

Bruker SMART APEXII CCD area-detector diffractometer
Absorption correction: multi-scan (*SADABS*; Bruker, 2005 ▶) *T*_min_ = 0.832, *T*_max_ = 0.862
12887 measured reflections
4187 independent reflections
2889 reflections with *I* > 2σ(*I*)
*R*_int_ = 0.083

#### Refinement

*R*[*F* ^2^ > 2σ(*F* ^2^)] = 0.044
*wR*(*F* ^2^) = 0.079
*S* = 0.86
4187 reflections
310 parameters
1 restraint
H-atom parameters constrained
Δρ_max_ = 0.59 e Å^−3^
Δρ_min_ = −0.22 e Å^−3^
Absolute structure: Flack (1983 ▶), 1898 Friedel pairs
Flack parameter: 0.584 (14)

### 

Data collection: *APEX2* (Bruker, 2005 ▶); cell refinement: *SAINT* (Bruker, 2005 ▶); data reduction: *SAINT*; program(s) used to solve structure: *SHELXS97* (Sheldrick, 2008 ▶); program(s) used to refine structure: *SHELXL97* (Sheldrick, 2008 ▶); molecular graphics: *SHELXTL* (Sheldrick, 2008 ▶); software used to prepare material for publication: *SHELXTL*.

## Supplementary Material

Crystal structure: contains datablocks I, global. DOI: 10.1107/S1600536809039191/fj2245sup1.cif

Structure factors: contains datablocks I. DOI: 10.1107/S1600536809039191/fj2245Isup2.hkl

Additional supplementary materials:  crystallographic information; 3D view; checkCIF report

## Figures and Tables

**Table 1:** Hydrogen-bond geometry (Å, °)

*D*—H⋯*A*	*D*—H	H⋯*A*	*D*⋯*A*	*D*—H⋯*A*
O1*W*—H1*WB*⋯O2	0.85	1.85	2.668 (4)	162
O1*W*—H1*WA*⋯O4^i^	0.85	2.06	2.821 (4)	149

Symmetry code: (i) 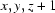.

**Fig. 1.**
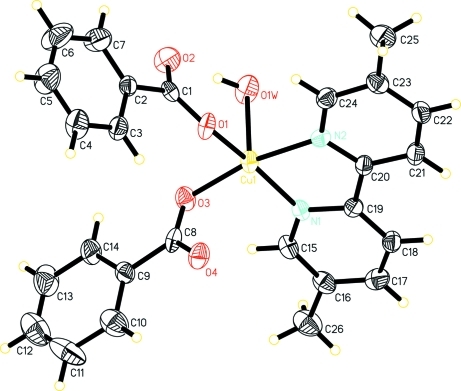
The molecular structure of the title compound with the atom-labelling scheme. Displacement ellipsoids are drawn at the 30% probability level. H atoms are presented as small spheres of arbitrary radius.

**Fig. 2.**
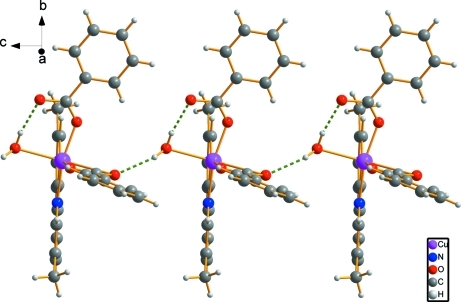
Crystal packing of the title complound. Hydrogen-bond interactions are drawn with dashed lines.

## supplementary crystallographic information

### Comment

As a contribution to structural characterization of 5,5'-dimethyl-2,2'-bipyridine complexes (Zhao *et al.* 2009; Schubert, Eschbaumer *et al.* 1999; Schubert, Hochwimmer *et al.* 1999; Yang *et al.*, 2001) we present here the crystal structure of the title complex, [Cu*L*_2_*L*'(H_2_O)](*L*=benzoate, *L*'=5,5'-dimethyl-2,2'-bipyridine).

In the complex, the Cu^2+^ ion is pentacoordinated, with two N atoms of 5,5'-dimethyl-2,2'-bipyridine and two O atoms of two benzoate ligands in the basal plane and the O atom of water molecule completing the square-pyramidal geometry from the apical site (Fig. 1). The atoms N1, N2, O1 and O3 are nearly coplanar, and the Cu atom is displaced by 0.2071 (6) Å from this plane towards the apical O atom.

With O—H···O hydrogen bonds (Table 1), a one-dimensional chain is formed as shown in Fig.2.

### Experimental

The title complound was synthesized hydrothermally in a Teflon-lined autoclave (25 ml) by heating a mixture of 5,5'-dimethyl-2,2'-bipyridine (0.2 mmol), benzoic acid (0.4 mmol) and CuSO_4_.5H_2_O (0.2 mmol) in water (10 ml) at 393 K for 3 d. Crystals suitable for X-ray analysis were obtained.

### Refinement

All H atoms were included in calculated positions, with C—H bond lengths fixed at 0.96 Å (methyl CH_3_), 0.93Å (aryl group) and O—H = 0.85 Å and were refined in the riding-model approximation. *U*_iso_(H) values were calculated at 1.5 *U*_eq_(C) for methyl groups and 1.2 *U*_eq_(C) otherwise. The refined value of Flack parameter of 0.584 (14) suggests that the crystal studied was a racemic twin.

### Crystal data

**Table:**

[Cu(C_7_H_5_O_2_)_2_(C_12_H_12_N_2_)(H_2_O)]	*F*(000) = 4208
*M**_r_* = 508.01	*D*_x_ = 1.412 Mg m^−^^3^
Orthorhombic, *F**d**d*2	Mo *K*α radiation, λ = 0.71073 Å
Hall symbol: F 2 -2d	Cell parameters from 2251 reflections
*a* = 36.033 (6) Å	θ = 2.3–27.4°
*b* = 37.681 (6) Å	µ = 0.95 mm^−^^1^
*c* = 7.0402 (12) Å	*T* = 296 K
*V* = 9559 (3) Å^3^	Block, blue
*Z* = 16	0.20 × 0.18 × 0.16 mm

### Data collection

**Table:**

Bruker SMART APEXII CCD area-detector diffractometer	4187 independent reflections
Radiation source: fine-focus sealed tube	2889 reflections with *I* > 2σ(*I*)
graphite	*R*_int_ = 0.083
φ and ω scans	θ_max_ = 25.0°, θ_min_ = 1.6°
Absorption correction: multi-scan (*SADABS*; Bruker, 2005)	*h* = −34→42
*T*_min_ = 0.832, *T*_max_ = 0.862	*k* = −44→43
12887 measured reflections	*l* = −8→8

### Refinement

**Table:**

Refinement on *F*^2^	Secondary atom site location: difference Fourier map
Least-squares matrix: full	Hydrogen site location: inferred from neighbouring sites
*R*[*F*^2^ > 2σ(*F*^2^)] = 0.044	H-atom parameters constrained
*wR*(*F*^2^) = 0.079	*w* = 1/[σ^2^(*F*_o_^2^) + (0.0232*P*)^2^] where *P* = (*F*_o_^2^ + 2*F*_c_^2^)/3
*S* = 0.86	(Δ/σ)_max_ = 0.001
4187 reflections	Δρ_max_ = 0.59 e Å^−^^3^
310 parameters	Δρ_min_ = −0.22 e Å^−^^3^
1 restraint	Absolute structure: Flack (1983), 1898 Friedel pairs
Primary atom site location: structure-invariant direct methods	Flack parameter: 0.584 (14)

### Special details

**Table:**

Geometry. All e.s.d.'s (except the e.s.d. in the dihedral angle between two l.s. planes) are estimated using the full covariance matrix. The cell e.s.d.'s are taken into account individually in the estimation of e.s.d.'s in distances, angles and torsion angles; correlations between e.s.d.'s in cell parameters are only used when they are defined by crystal symmetry. An approximate (isotropic) treatment of cell e.s.d.'s is used for estimating e.s.d.'s involving l.s. planes.
Refinement. Refinement of *F*^2^ against ALL reflections. The weighted *R*-factor *wR* and goodness of fit *S* are based on *F*^2^, conventional *R*-factors *R* are based on *F*, with *F* set to zero for negative *F*^2^. The threshold expression of *F*^2^ > σ(*F*^2^) is used only for calculating *R*-factors(gt) *etc*. and is not relevant to the choice of reflections for refinement. *R*-factors based on *F*^2^ are statistically about twice as large as those based on *F*, and *R*- factors based on ALL data will be even larger.

### Fractional atomic coordinates and isotropic or equivalent isotropic displacement parameters (Å^2^)

**Table:**

	*x*	*y*	*z*	*U*_iso_*/*U*_eq_	
Cu1	0.289268 (13)	0.075081 (13)	0.86862 (8)	0.04372 (16)	
N1	0.29272 (9)	0.02219 (8)	0.9019 (5)	0.0390 (9)	
N2	0.23481 (8)	0.06306 (8)	0.8887 (6)	0.0387 (8)	
O1	0.27971 (8)	0.12241 (8)	0.7744 (4)	0.0525 (9)	
O2	0.29910 (9)	0.15568 (9)	1.0158 (5)	0.0675 (10)	
O3	0.34227 (7)	0.07939 (8)	0.8118 (4)	0.0501 (9)	
O4	0.32544 (8)	0.06623 (8)	0.5189 (4)	0.0576 (9)	
C1	0.29069 (11)	0.15122 (12)	0.8432 (8)	0.0412 (11)	
C2	0.29381 (12)	0.18240 (12)	0.7136 (7)	0.0402 (11)	
C3	0.29679 (13)	0.17730 (13)	0.5216 (8)	0.0530 (14)	
H3	0.2958	0.1545	0.4718	0.064*	
C4	0.30136 (13)	0.20622 (14)	0.4007 (8)	0.0681 (16)	
H4	0.3040	0.2025	0.2709	0.082*	
C5	0.30205 (16)	0.23955 (16)	0.4693 (9)	0.0833 (19)	
H5	0.3049	0.2588	0.3877	0.100*	
C6	0.29856 (18)	0.24474 (14)	0.6595 (10)	0.103 (2)	
H6	0.2990	0.2677	0.7077	0.123*	
C7	0.29437 (15)	0.21623 (14)	0.7835 (7)	0.0730 (16)	
H7	0.2920	0.2202	0.9133	0.088*	
C8	0.34916 (11)	0.07366 (10)	0.6394 (8)	0.0440 (11)	
C9	0.38971 (12)	0.07565 (11)	0.5812 (7)	0.0466 (12)	
C10	0.40021 (16)	0.06443 (13)	0.4021 (9)	0.0782 (16)	
H10	0.3829	0.0554	0.3168	0.094*	
C11	0.4386 (2)	0.06724 (17)	0.3528 (11)	0.101 (2)	
H11	0.4467	0.0596	0.2342	0.121*	
C12	0.46305 (19)	0.08087 (18)	0.4772 (11)	0.099 (2)	
H12	0.4878	0.0829	0.4420	0.119*	
C13	0.45266 (15)	0.09165 (15)	0.6505 (12)	0.088 (2)	
H13	0.4701	0.1007	0.7350	0.105*	
C14	0.41569 (13)	0.08922 (12)	0.7028 (8)	0.0634 (15)	
H14	0.4084	0.0969	0.8226	0.076*	
C15	0.32335 (11)	0.00233 (12)	0.9057 (7)	0.0493 (12)	
H15	0.3460	0.0142	0.9024	0.059*	
C16	0.32444 (13)	−0.03421 (12)	0.9142 (6)	0.0503 (13)	
C17	0.29028 (15)	−0.05082 (12)	0.9140 (7)	0.0554 (14)	
H17	0.2891	−0.0755	0.9163	0.066*	
C18	0.25819 (12)	−0.03145 (11)	0.9103 (6)	0.0463 (12)	
H18	0.2354	−0.0430	0.9119	0.056*	
C19	0.25954 (11)	0.00489 (11)	0.9043 (6)	0.0380 (10)	
C20	0.22719 (11)	0.02858 (10)	0.9028 (6)	0.0382 (10)	
C21	0.19083 (12)	0.01653 (12)	0.9156 (6)	0.0478 (12)	
H21	0.1858	−0.0076	0.9220	0.057*	
C22	0.16258 (12)	0.04087 (12)	0.9184 (6)	0.0507 (13)	
H22	0.1382	0.0330	0.9271	0.061*	
C23	0.16969 (12)	0.07650 (13)	0.9088 (7)	0.0504 (13)	
C24	0.20738 (12)	0.08629 (10)	0.8924 (7)	0.0476 (11)	
H24	0.2132	0.1103	0.8837	0.057*	
C25	0.13916 (12)	0.10390 (12)	0.9142 (8)	0.0735 (16)	
H25A	0.1293	0.1071	0.7888	0.110*	
H25B	0.1490	0.1260	0.9594	0.110*	
H25C	0.1198	0.0960	0.9980	0.110*	
C26	0.35995 (13)	−0.05492 (13)	0.9193 (8)	0.0753 (17)	
H26A	0.3771	−0.0450	0.8296	0.113*	
H26B	0.3550	−0.0792	0.8869	0.113*	
H26C	0.3704	−0.0538	1.0445	0.113*	
O1W	0.29338 (8)	0.09216 (8)	1.1806 (4)	0.0668 (9)	
H1WA	0.3103	0.0852	1.2558	0.080*	
H1WB	0.2988	0.1131	1.1452	0.080*	

### Atomic displacement parameters (Å^2^)

**Table:**

	*U*^11^	*U*^22^	*U*^33^	*U*^12^	*U*^13^	*U*^23^
Cu1	0.0381 (3)	0.0476 (3)	0.0454 (3)	−0.0093 (3)	−0.0019 (3)	0.0048 (3)
N1	0.034 (2)	0.049 (2)	0.034 (2)	−0.0058 (18)	0.0006 (18)	0.002 (2)
N2	0.041 (2)	0.037 (2)	0.038 (2)	0.0015 (15)	−0.002 (2)	−0.005 (2)
O1	0.051 (2)	0.046 (2)	0.060 (2)	−0.0196 (15)	−0.0110 (16)	0.0072 (16)
O2	0.089 (3)	0.061 (2)	0.052 (2)	−0.0008 (18)	−0.002 (2)	0.0060 (18)
O3	0.043 (2)	0.065 (2)	0.043 (2)	−0.0107 (14)	−0.0032 (14)	0.0033 (17)
O4	0.054 (2)	0.060 (2)	0.058 (2)	−0.0155 (16)	−0.0136 (18)	−0.0003 (17)
C1	0.026 (2)	0.052 (3)	0.045 (3)	−0.002 (2)	0.007 (2)	0.003 (3)
C2	0.037 (3)	0.035 (3)	0.048 (3)	−0.002 (2)	0.004 (2)	0.005 (2)
C3	0.054 (4)	0.053 (4)	0.052 (4)	−0.016 (2)	−0.003 (3)	−0.001 (3)
C4	0.084 (4)	0.074 (4)	0.046 (4)	−0.020 (3)	−0.011 (3)	0.019 (3)
C5	0.116 (5)	0.059 (4)	0.074 (5)	−0.010 (3)	0.001 (3)	0.024 (4)
C6	0.183 (7)	0.044 (4)	0.080 (5)	−0.009 (3)	0.015 (5)	0.000 (4)
C7	0.111 (5)	0.055 (4)	0.053 (4)	0.002 (3)	0.011 (3)	0.003 (3)
C8	0.042 (3)	0.037 (3)	0.053 (3)	−0.0150 (19)	−0.012 (3)	0.007 (3)
C9	0.050 (3)	0.037 (3)	0.052 (3)	0.003 (2)	0.011 (2)	0.013 (3)
C10	0.077 (4)	0.086 (4)	0.072 (4)	0.013 (3)	0.010 (3)	0.016 (4)
C11	0.102 (6)	0.128 (6)	0.072 (5)	0.053 (4)	0.041 (5)	0.032 (5)
C12	0.080 (5)	0.112 (6)	0.105 (7)	0.019 (4)	0.023 (5)	0.042 (6)
C13	0.050 (4)	0.086 (4)	0.127 (7)	−0.003 (3)	−0.006 (4)	0.007 (4)
C14	0.036 (3)	0.069 (4)	0.086 (4)	−0.008 (2)	0.001 (3)	−0.005 (3)
C15	0.039 (3)	0.058 (3)	0.051 (3)	−0.002 (2)	0.004 (2)	0.011 (3)
C16	0.053 (3)	0.058 (3)	0.040 (3)	0.008 (2)	0.007 (2)	0.015 (3)
C17	0.082 (4)	0.040 (3)	0.043 (4)	0.005 (3)	0.005 (3)	0.007 (2)
C18	0.051 (3)	0.044 (3)	0.044 (3)	−0.010 (2)	0.004 (2)	0.001 (3)
C19	0.051 (3)	0.039 (3)	0.024 (3)	−0.006 (2)	−0.001 (2)	0.002 (2)
C20	0.042 (3)	0.042 (3)	0.030 (3)	−0.010 (2)	−0.001 (2)	0.000 (2)
C21	0.048 (3)	0.047 (3)	0.048 (3)	−0.014 (2)	0.001 (2)	0.001 (2)
C22	0.042 (3)	0.058 (3)	0.052 (4)	−0.011 (2)	0.005 (2)	−0.005 (3)
C23	0.043 (3)	0.062 (3)	0.046 (4)	0.001 (2)	0.000 (2)	−0.002 (3)
C24	0.055 (3)	0.045 (3)	0.044 (3)	−0.005 (2)	−0.001 (3)	−0.003 (3)
C25	0.055 (3)	0.073 (4)	0.093 (4)	0.007 (3)	0.010 (3)	−0.011 (3)
C26	0.069 (4)	0.079 (4)	0.078 (4)	0.024 (3)	0.011 (3)	0.017 (3)
O1W	0.087 (3)	0.067 (2)	0.047 (2)	−0.0211 (17)	−0.0145 (16)	0.0103 (17)

### Geometric parameters (Å, °)

**Table:**

Cu1—O1	1.934 (3)	C11—H11	0.9300
Cu1—O3	1.958 (3)	C12—C13	1.339 (9)
Cu1—N1	2.011 (3)	C12—H12	0.9300
Cu1—N2	2.019 (3)	C13—C14	1.385 (6)
Cu1—O1W	2.294 (3)	C13—H13	0.9300
N1—C15	1.334 (5)	C14—H14	0.9300
N1—C19	1.362 (5)	C15—C16	1.379 (5)
N2—C24	1.321 (5)	C15—H15	0.9300
N2—C20	1.332 (4)	C16—C17	1.381 (6)
O1—C1	1.253 (5)	C16—C26	1.499 (5)
O2—C1	1.263 (6)	C17—C18	1.368 (5)
O3—C8	1.258 (5)	C17—H17	0.9300
O4—C8	1.236 (5)	C18—C19	1.371 (5)
C1—C2	1.492 (6)	C18—H18	0.9300
C2—C7	1.367 (6)	C19—C20	1.468 (6)
C2—C3	1.369 (6)	C20—C21	1.390 (5)
C3—C4	1.392 (6)	C21—C22	1.370 (6)
C3—H3	0.9300	C21—H21	0.9300
C4—C5	1.346 (7)	C22—C23	1.369 (6)
C4—H4	0.9300	C22—H22	0.9300
C5—C6	1.359 (8)	C23—C24	1.412 (5)
C5—H5	0.9300	C23—C25	1.509 (6)
C6—C7	1.392 (7)	C24—H24	0.9300
C6—H6	0.9300	C25—H25A	0.9600
C7—H7	0.9300	C25—H25B	0.9600
C8—C9	1.519 (6)	C25—H25C	0.9600
C9—C14	1.368 (6)	C26—H26A	0.9600
C9—C10	1.382 (7)	C26—H26B	0.9600
C10—C11	1.431 (7)	C26—H26C	0.9600
C10—H10	0.9300	O1W—H1WA	0.8500
C11—C12	1.344 (9)	O1W—H1WB	0.8501
			
O1—Cu1—O3	91.56 (12)	C11—C12—H12	119.1
O1—Cu1—N1	164.83 (14)	C12—C13—C14	119.4 (7)
O3—Cu1—N1	92.61 (13)	C12—C13—H13	120.3
O1—Cu1—N2	93.32 (12)	C14—C13—H13	120.3
O3—Cu1—N2	168.80 (15)	C9—C14—C13	121.1 (5)
N1—Cu1—N2	80.19 (13)	C9—C14—H14	119.5
O1—Cu1—O1W	94.67 (12)	C13—C14—H14	119.5
O3—Cu1—O1W	96.28 (12)	N1—C15—C16	125.8 (4)
N1—Cu1—O1W	99.37 (13)	N1—C15—H15	117.1
N2—Cu1—O1W	93.37 (13)	C16—C15—H15	117.1
C15—N1—C19	117.2 (4)	C15—C16—C17	115.3 (4)
C15—N1—Cu1	127.6 (3)	C15—C16—C26	123.0 (4)
C19—N1—Cu1	114.9 (3)	C17—C16—C26	121.7 (5)
C24—N2—C20	119.4 (3)	C18—C17—C16	120.8 (4)
C24—N2—Cu1	125.5 (3)	C18—C17—H17	119.6
C20—N2—Cu1	115.1 (3)	C16—C17—H17	119.6
C1—O1—Cu1	127.6 (3)	C17—C18—C19	120.2 (4)
C8—O3—Cu1	112.1 (3)	C17—C18—H18	119.9
O1—C1—O2	124.3 (4)	C19—C18—H18	119.9
O1—C1—C2	118.0 (5)	N1—C19—C18	120.6 (4)
O2—C1—C2	117.7 (4)	N1—C19—C20	113.9 (4)
C7—C2—C3	119.0 (5)	C18—C19—C20	125.4 (4)
C7—C2—C1	121.0 (5)	N2—C20—C21	121.2 (4)
C3—C2—C1	119.9 (5)	N2—C20—C19	115.5 (4)
C2—C3—C4	120.2 (5)	C21—C20—C19	123.3 (4)
C2—C3—H3	119.9	C22—C21—C20	118.9 (4)
C4—C3—H3	119.9	C22—C21—H21	120.6
C5—C4—C3	120.9 (5)	C20—C21—H21	120.6
C5—C4—H4	119.6	C23—C22—C21	121.1 (4)
C3—C4—H4	119.6	C23—C22—H22	119.4
C4—C5—C6	119.1 (5)	C21—C22—H22	119.4
C4—C5—H5	120.5	C22—C23—C24	116.1 (4)
C6—C5—H5	120.5	C22—C23—C25	122.2 (4)
C5—C6—C7	121.1 (6)	C24—C23—C25	121.6 (4)
C5—C6—H6	119.5	N2—C24—C23	123.2 (4)
C7—C6—H6	119.5	N2—C24—H24	118.4
C2—C7—C6	119.7 (5)	C23—C24—H24	118.4
C2—C7—H7	120.1	C23—C25—H25A	109.5
C6—C7—H7	120.1	C23—C25—H25B	109.5
O4—C8—O3	124.4 (4)	H25A—C25—H25B	109.5
O4—C8—C9	119.4 (5)	C23—C25—H25C	109.5
O3—C8—C9	116.2 (4)	H25A—C25—H25C	109.5
C14—C9—C10	119.9 (4)	H25B—C25—H25C	109.5
C14—C9—C8	120.5 (4)	C16—C26—H26A	109.5
C10—C9—C8	119.6 (5)	C16—C26—H26B	109.5
C9—C10—C11	117.6 (6)	H26A—C26—H26B	109.5
C9—C10—H10	121.2	C16—C26—H26C	109.5
C11—C10—H10	121.2	H26A—C26—H26C	109.5
C12—C11—C10	120.2 (7)	H26B—C26—H26C	109.5
C12—C11—H11	119.9	Cu1—O1W—H1WA	123.8
C10—C11—H11	119.9	Cu1—O1W—H1WB	89.7
C13—C12—C11	121.8 (7)	H1WA—O1W—H1WB	107.7
C13—C12—H12	119.1		
			
O1—Cu1—N1—C15	113.7 (5)	O3—C8—C9—C14	−11.4 (6)
O3—Cu1—N1—C15	7.9 (4)	O4—C8—C9—C10	−8.9 (6)
N2—Cu1—N1—C15	179.3 (4)	O3—C8—C9—C10	170.1 (4)
O1W—Cu1—N1—C15	−88.9 (4)	C14—C9—C10—C11	0.7 (7)
O1—Cu1—N1—C19	−60.3 (7)	C8—C9—C10—C11	179.2 (4)
O3—Cu1—N1—C19	−166.1 (3)	C9—C10—C11—C12	−1.0 (8)
N2—Cu1—N1—C19	5.2 (3)	C10—C11—C12—C13	1.1 (10)
O1W—Cu1—N1—C19	97.1 (3)	C11—C12—C13—C14	−0.9 (10)
O1—Cu1—N2—C24	−18.4 (4)	C10—C9—C14—C13	−0.6 (7)
O3—Cu1—N2—C24	−134.1 (6)	C8—C9—C14—C13	−179.1 (4)
N1—Cu1—N2—C24	175.4 (4)	C12—C13—C14—C9	0.7 (8)
O1W—Cu1—N2—C24	76.5 (4)	C19—N1—C15—C16	−0.8 (7)
O1—Cu1—N2—C20	163.0 (3)	Cu1—N1—C15—C16	−174.7 (4)
O3—Cu1—N2—C20	47.3 (9)	N1—C15—C16—C17	1.6 (7)
N1—Cu1—N2—C20	−3.2 (3)	N1—C15—C16—C26	−179.7 (4)
O1W—Cu1—N2—C20	−102.2 (3)	C15—C16—C17—C18	−1.6 (7)
O3—Cu1—O1—C1	−66.4 (4)	C26—C16—C17—C18	179.7 (4)
N1—Cu1—O1—C1	−172.3 (5)	C16—C17—C18—C19	0.9 (7)
N2—Cu1—O1—C1	123.7 (4)	C15—N1—C19—C18	−0.1 (6)
O1W—Cu1—O1—C1	30.1 (4)	Cu1—N1—C19—C18	174.6 (3)
O1—Cu1—O3—C8	−80.8 (3)	C15—N1—C19—C20	179.1 (4)
N1—Cu1—O3—C8	84.6 (3)	Cu1—N1—C19—C20	−6.3 (5)
N2—Cu1—O3—C8	35.0 (8)	C17—C18—C19—N1	0.0 (7)
O1W—Cu1—O3—C8	−175.7 (3)	C17—C18—C19—C20	−179.0 (4)
Cu1—O1—C1—O2	−25.0 (6)	C24—N2—C20—C21	2.0 (6)
Cu1—O1—C1—C2	155.5 (3)	Cu1—N2—C20—C21	−179.3 (3)
O1—C1—C2—C7	159.8 (4)	C24—N2—C20—C19	−177.9 (4)
O2—C1—C2—C7	−19.7 (7)	Cu1—N2—C20—C19	0.8 (5)
O1—C1—C2—C3	−21.3 (7)	N1—C19—C20—N2	3.6 (6)
O2—C1—C2—C3	159.2 (4)	C18—C19—C20—N2	−177.3 (4)
C7—C2—C3—C4	1.8 (8)	N1—C19—C20—C21	−176.3 (4)
C1—C2—C3—C4	−177.1 (4)	C18—C19—C20—C21	2.8 (7)
C2—C3—C4—C5	−1.6 (8)	N2—C20—C21—C22	−1.7 (6)
C3—C4—C5—C6	0.6 (9)	C19—C20—C21—C22	178.1 (4)
C4—C5—C6—C7	0.1 (10)	C20—C21—C22—C23	0.1 (7)
C3—C2—C7—C6	−1.1 (8)	C21—C22—C23—C24	1.1 (7)
C1—C2—C7—C6	177.9 (5)	C21—C22—C23—C25	−179.2 (5)
C5—C6—C7—C2	0.1 (9)	C20—N2—C24—C23	−0.7 (7)
Cu1—O3—C8—O4	0.1 (5)	Cu1—N2—C24—C23	−179.2 (4)
Cu1—O3—C8—C9	−178.9 (2)	C22—C23—C24—N2	−0.9 (7)
O4—C8—C9—C14	169.5 (4)	C25—C23—C24—N2	179.4 (5)

### Hydrogen-bond geometry (Å, °)

**Table:**

*D*—H···*A*	*D*—H	H···*A*	*D*···*A*	*D*—H···*A*
O1W—H1WB···O2	0.85	1.85	2.668 (4)	162
O1W—H1WA···O4^i^	0.85	2.06	2.821 (4)	149

Symmetry codes: (i) *x*, *y*, *z*+1.
